# Nuclear Immunolocalization of Hexamerins in the Fat Body of Metamorphosing Honey Bees

**DOI:** 10.3390/insects3041039

**Published:** 2012-10-22

**Authors:** Juliana Ramos Martins, Márcia Maria Gentile Bitondi

**Affiliations:** 1 Departamento de Genética, Faculdade de Medicina de Ribeirão Preto, Universidade de São Paulo, Ribeirão Preto, SP 14049-900, Brasil; E-Mail: julianarmartins@usp.br; 2 Departamento de Biologia, Faculdade de Filosofia, Ciências e Letras de Ribeirão Preto, Universidade de São Paulo, Ribeirão Preto, SP 14040-901, Brasil

**Keywords:** hexamerin, HEX 70a, HEX 70b, HEX 70c, HEX 110, fat body, metamorphosis, honey bee, *Apis mellifera*

## Abstract

Hexamerins are storage proteins with primordial functions in insect metamorphosis. They are actively secreted by the larval fat body and stored in the hemolymph. During metamorphosis, they return to the fat body to be processed. For decades, these proteins were thought to exclusively function as an amino acid source for tissue reconstruction during the non-feeding pupal and pharate adult stages and, in some species, for egg production. Recently, new findings have linked the hexamerins to caste polyphenism and gonad development in social insects. To explore the roles of hexamerins during the honey bee metamorphosis, we used specific antibodies in expression analysis by western blot, *in situ* immunolocalization by confocal laser-scanning microscopy and *in vivo* injections to lower their endogenous levels. Our expression analysis highlighted the changing expression patterns in the fat body and hemolymph during development, which is consistent with the temporal dynamics of hexamerin secretion, storage and depletion. Confocal microscopy showed hexamerin expression in the cytoplasm of both types of fat body cells, trophocytes and oenocytes. Notably, hexamerin foci were also found in the nuclei of these cells, thus confirming our western blot analysis of fat body nuclear-enriched fractions. We also observed that the decrease in soluble hexamerins in antibody-treated pharate adults led to a precocious adult ecdysis, perhaps in response to the lack (or decrease) in hexamerin-derived amino acids. Taken together, these findings indicate that hexamerins have other functions in addition to their well-established role as amino acid sources for development.

## 1. Introduction

The fat body is an abundant tissue in the insect abdomen where it is localized subjacent to the epidermis (parietal fat body) and around the gut (visceral fat body). It is also found in association with the gonads, muscles and regions outside the abdomen [1]. The trophocyte, the basic fat body cell type, the oenocyte, another important cell, and other less common cells in some insect species, such as the urocytes and mycetocytes, have long been identified as the structural components of the fat body [2]. The fat body is bathed in the hemolymph, which is important for optimizing the secretion and uptake of molecules in spite of the basal lamina that interfaces between the tissue and the circulating fluid [2].

The fat body has a primordial role in the intermediary metabolism. During the larval stage, it is actively engaged in the metabolism of lipids and carbohydrates, and in the synthesis and secretion of proteins, which are stored in large quantities in the hemolymph [1,3]. Hemolymph storage proteins were first identified by Munn *et al.* [4,5,6] and Munn and Greville [7] in *Calliphora*. Wyatt and Pan [8] later identified these proteins in the hemolymph of several insects, thus confirming their general occurrence. The storage proteins have also been referred to as larval serum proteins in the literature. Some storage proteins have even been named according to the insect genus where they were identified; for example, calliphorin (from *Calliphora*), manducin (from *Manduca*) and others [9,10]. The name hexamerins, which has been privileged, refers to their hexameric structure.

The diverse amino acid compositions of the hexamerins have been used to classify them. For example, arylphorins are named for their high content of aromatic amino acids; there are also methionine-rich hexamerins and other types [10]. Phylogenetically, the hexamerins belong to the hemocyanin family; although they have lost the ability to bind and transport oxygen [11].

The hexamerins are mainly synthesized by the larval fat body and are secreted in the hemolymph. In general, the titer of these proteins in the hemolymph is highest at the end of the larval feeding stage, but the titer then progressively decreases due to their uptake by the fat body during and after metamorphosis. Munn and Greville [7] originally proposed that the hexamerins are the protein reserves in the fat body during the development of the post-metamorphic non-feeding stages (pupae and pharate adults). In *Calpodes* [12] and in *Hyalophora cecropia* [13], hexamerins form membrane-bound protein storage granules in the fat body cell cytoplasm. Storage proteins were also isolated from the fat body of *Bombyx mori* [14], presumably from protein granules, and the uptake of hemolymph proteins into fat body granules was demonstrated in *Drosophila* [15].

Hexamerins may have other functions in addition to being storage proteins. In grasshoppers, they may play a role as hemolymph juvenile hormone transporters [16,17,18]. It was also demonstrated that hexamerins interact with other proteins in a multiprotein complex engaged in sequestration and transport of juvenile hormone, thus regulating its levels and actions [19], including the action on caste determination in social insects. In the termite *Reticulitermes flavipes*, a hexamerin has been associated with the regulation of the juvenile hormone-dependent soldier caste phenotype [20,21,22,23]. 

To our knowledge, Ryan *et al.* [24] were the first to characterize a hexamerin in the honey bee. They identified a 74 kDa hexamerin subunit in the larval hemolymph using an antiserum against a *M. sexta* arylphorin. Years later, Danty *et al.* [25] analyzed the expression of the 74 kDa hexamerin subunit and the other three hexamerin subunits of the honey bee. The respective N-termini were sequenced, and they were named HEX 70a, HEX 70b, HEX 70c and HEX 110, corresponding to the approximate molecular mass determined by SDS-PAGE migration. Our laboratory further characterized the cDNAs encoding all four hexamerin subunits, their transcriptional profiles in the developing fat body, their regulation by morphogenetic hormones and their expression dependent on nutritional intake. We also looked for potential regulatory motifs shared by the four hexamerin genes in the 5' upstream control regions, which could indicate that they are co-regulated [26,27,28,29]. In addition, we investigated the expression of the honey bee hexamerins outside the fat body. Interestingly, transcripts for HEX 110, HEX 70a and HEX 70b were detected by qRT-PCR in the developing female and male gonads [29]. Furthermore, the presence of HEX 70a was confirmed in the developing ovaries and testes by antibody staining and confocal microscopy. Surprisingly, foci of HEX 70a were found not only in the cytoplasm of the gonadal germ and somatic cells, but also in their nuclei [30]. These results indicated that, at least in the gonads, the hexamerins have alternative roles. The presence of foci of HEX 70a in the nuclei of gonadal cells prompted us to look for the localization of hexamerins in the fat body cells. We also compared the temporal dynamics of hexamerins expression in the fat body and hemolymph. In hopes of highlighting the importance of hexamerins in honey bee post-metamorphic development, we lowered their levels in pharate adults and examined the survival and timing of adult ecdysis.

## 2. Experimental Section

### 2.1. Bee Sampling

Honey bee workers (*Apis mellifera*, Africanized) in different developmental stages (fifth instar larvae, pharate pupae, pupae and pharate adults) and adults (newly-emerged, nurses and foragers) were collected from hives maintained at the apiary of the University of São Paulo in Ribeirão Preto, SP, Brazil. 

### 2.2. Sample Preparation for SDS-PAGE and Western Blot

Hemolymph from individual bees in each developmental phase was collected from a small lateral incision at the abdominal tergites, using microcapillary tubes previously washed in phenylthiourea solution in water. The hemolymph samples (one sample per developmental stage) were mixed with protease inhibitors cocktail (Protease Inhibitor Cocktail Tablets—Roche Applied Science) at the proportion of 10:1 (v/v), and centrifuged at 2,000 × *g* for 1 min at 4 °C. The supernatants were used for protein separation by SDS-PAGE and hexamerin identification by western blot and reaction with the specific anti-hexamerin antibodies. 

The fat body was quickly dissected from the same bees from which the hemolymph was collected, placed in microtubes, frozen in liquid nitrogen, powdered with the aid of a small glass pestle, and centrifuged at 10,000 × *g* for 10 min at 4 °C. The supernatants were used for SDS-PAGE followed by western blot and membrane incubation with each anti-hexamerin antibody. 

Nuclear fractions from the fat body of pooled fifth instar larvae at the spinning phase (L5S) were obtained by using a procedure based on Kirankumar *et al.* [31]. After being dissected in PBS_1_ (137 mM NaCl, 2.7 mM KCl, 10 mM Na_2_HPO_4_, 1.7 mM KH_2_PO_4_, pH 7.4), the fat body was rinsed three times in PBS_1_ and gently centrifuged at 250 × *g* for 15 min at 4 °C in 1.5 mL PBS_1_ containing 25 µL protease inhibitors cocktail. The pellet was resuspended in 1 mL HEPES buffer (5 mM HEPES pH 8.5 containing 0.1 mM CaCl_2_ and 10 µL protease inhibitors cocktail), homogenized by passing through a 13 × 0.45 mm Injex^TM^ needle 10 times and centrifuged at 1,000 × *g* for 5 min at 4 °C. Once again, the pellet was resuspended in 150 µL urea/Chaps buffer (8 M urea and 2% Chaps) containing 2.5 µL protease inhibitors cocktail, sonicated (ultrasonic equipment T14, 40 kHz, Thornton Electronica Ltda, São Paulo, Brazil) (three cycles of 5 min on ice followed by 5 min under vortex) and centrifuged at 20,000 × *g* for 30 min at 4 °C. The supernatant served as the source of crude nuclear proteins. 

Total protein was quantified [32] in all the hemolymph and fat body supernatants using bovine serum albumin in standard curves. Samples containing 5 µg of total protein were used for SDS-PAGE and western blot.

SDS-PAGE [33] was carried out at 15–20 mA and 4 °C using 7.5% polyacrylamide gels measuring 100 × 120 × 0.9 mm. Following electrophoresis, the proteins were transferred to nitrocellulose membranes (ImmunBlot^TM^ PVDF Membrane, Bio-Rad). The membranes were stained with 0.5% Ponceau in 1% acetic acid to check the migration of the sample proteins as well as the migration of the molecular mass markers (205, 116, 97.4, 66, 45 and 29 kDa, Sigma). Non-specific binding sites were blocked by incubating the membranes for 16 h in 10% non-fat dried milk in PBS_2_ (50 mM Tris, 80 mM NaCl, 2 mM CaCl_2_, pH 8.5). Hexamerins subunits were detected by incubating the membranes for 1 h at room temperature, with each hexamerin antibody diluted 1:5,000 (HEX 70a) or 1:1,000 (HEX 110, HEX 70b and HEX 70c) in 10% non-fat dried milk in PBS_2_. The membranes were washed thoroughly in 0.05% Tween 20 in PBS_1_ (TwPBS) and subsequently incubated for 1 h in a horseradish peroxidase labeled anti-rabbit IgG secondary antibody (GE Healthcare), diluted 1:12,000 in TwPBS. After washing in TwPBS and the detection was carried out by using the ECL System (ECL^TM^ Western Blotting Analysis System, GE Healthcare). 

### 2.3. Immunolocalization of the Hexamerins in the Fat Body Cells

Custom-made polyclonal hexamerins specific antibodies (Affinity BioReagents, Golden, CO, USA; Rheabiotech, Campinas, Brazil) were produced from the sequences NLYTKYHGQYP, SYKMHQKPYNKD, TFNLVENLDNYNDKEAVNEF, RNYDMESNMDMYKDKNVVQK of the HEX 110, HEX 70a, HEX 70b and HEX 70c subunits, respectively, predicted from the fully sequenced cDNAs [20,21]. These antibodies were used in whole mount preparations of the fat body from worker pharate pupae (PP phase) prepared as follows: the fat body was fixed for 20 min in 4% paraformaldehyde in PBS_1_, permeabilized with 0.1% Triton X-100 in PBS_1_ for 5 min (three washes) and blocked with 0.6% Triton X-100 in PBS_1_ containing 5% BSA for 5 min (five washes). This was followed by incubation in this same solution containing 10% goat serum for 1 h. The fat body was then reincubated with each hexamerin antibody at a concentration of 1:50 in 0.6% Triton X-100 in PBS_1_ plus 5% BSA and 10% goat serum for 16 h at 4 °C. This was followed by three washes of 5 min and five washes of 20 min in 0.6% Triton X-100 in PBS_1 _plus 5% BSA and subsequent incubation in Alexa Fluor 488 (1:200, Invitrogen) for 2 h at room temperature. The samples were rinsed again (three washes of 5 min and five washes of 20 min in 0.6% Triton X-100 in PBS_1 _containing 5% BSA). To stain the cell nuclei, the fat body was subsequently incubated for 15 min in 1 µg propidium iodide (Invitrogen) diluted in 500 µL of 0.6% Triton X-100 in PBS_1_. Propidium iodide is an intercalating molecule that binds DNA and RNA. After rinsing five times in 0.6% Triton X-100 in PBS_1_, the fat body was transferred to glass slides, mounted on glycerol 80% (Merck) and examined under a Leica TCS-SP5 confocal microscope (Leica Microsystems).

### 2.4. Injection of Antibodies against Hexamerins

Pupae (Pw phase) were collected from hives and maintained in an incubator at 34 °C and 80% relative humidity for 24 h before receiving an injection of 1 µL of an antibody (1 µg in 0.9% NaCl) or only 0.9% NaCl into the abdominal hemocoel. Four groups of 12 pupae were respectively injected with antibody against HEX 70a, HEX 70b, HEX 70c or HEX 110. Control groups were injected with 1 µL of 0.9% NaCl (8 pupae) or 1 µL of mouse IgG (1 µg in 0.9% NaCl) (6 pupae). The injected bees were maintained in the incubator, at the same conditions mentioned above, up to the adult ecdysis. The hemolymph or fat body was collected 4 h, 24 h and 129 h after the injection. Hemolymph and fat body were processed as described in the item 2.2 above. Total protein was quantified [32] in the supernatants, and samples containing 5 µg of total protein were used for SDS-PAGE and western blot analyses to attest the levels of soluble hexamerins. Survival, as well as the time elapsed to the adult ecdysis, were verified.

## 3. Results

### 3.1. Dynamics of Hexamerin Expression in the Hemolymph and Fat Body during and after Metamorphosis

**Figure 1:**
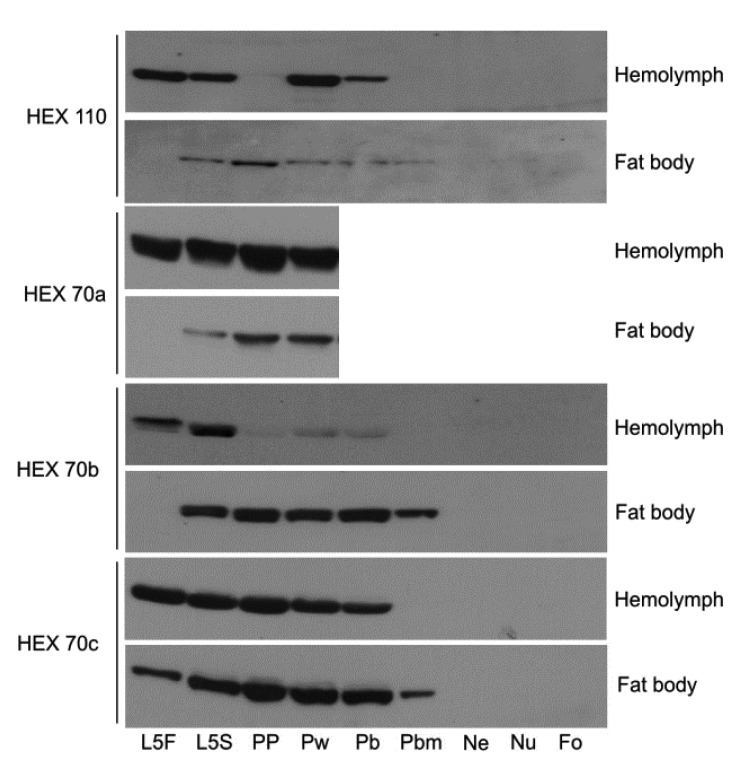
Western blot analysis of the four hexamerins (HEX 110, HEX 70a, HEX 70b and HEX 70c) in the hemolymph and fat body of developing *Apis mellifera* workers. L5F and L5S: feeding and spinning fifth instar larvae. PP: pharate pupa. Pw: pupa. Pb, Pbm: successive phases of pharate adults. NE: newly ecdysed adult. Nu: nurse bee. Fo: forager bee. The western blots showing HEX 70a in the fat body and in the hemolymph of pharate adults (Pb and Pbm phases) and adults (three days old Nu and 24 days old Fo) were shown previously (see Figure 2 in [28]).

We verified the expression of the four hexamerins in the hemolymph and fat body during precise developmental time points by western blotting (Figure 1). In fifth instar feeding larva (L5F), all hexamerins exist in a larger quantity in the hemolymph than in the fat body, indicating intense secretion to the hemolymph. During the next developmental phase, when the fifth instar spinning larva (L5S) prepares for the metamorphic molt, the abundance of all hexamerins increases in the fat body. Based on what is known about the exchange of hexamerins between the fat body and hemolymph, this increase may denote the resorption of hexamerins into the fat body, via sequestration from the hemolymph. From the L5S time point to the pharate adult phases (Pb, Pbm), the hexamerins still remain relatively abundant in the fat body, although HEX 110 is the less abundant. The abundance of all hexamerins in the fat body decreases to basal levels near the time of adult ecdysis, *i.e.*, at the end of the last pharate adult phase, Pbd (not shown in Figure 1). However, HEX 70a levels increase again in the adults, where this protein persists, even in foragers. The persistence of HEX 70a in the hemolymph of adult workers was first demonstrated by Coomassie Blue stained SDS-PAGE [25]. Since HEX 70a comigrates with the small lipophorin subunit (ApoLp-II) [34], we used the specific antibody to verify and confirm its persistence in adults [28]. This result was reinforced by the detection of HEX 70a transcripts in adult workers from emergence up to 30 days of adult life [28,29]. 

The depletion rate of HEX 70b, HEX 70c and HEX 110 in the hemolymph is variable; HEX 70b is depleted earlier. The depletion of hexamerins is consistent with their function as amino acid sources for pupal and pharate adult development. 

### 3.2. Detection of Hexamerins in Isolated Fat Body Cell Nuclei

**Figure 2:**
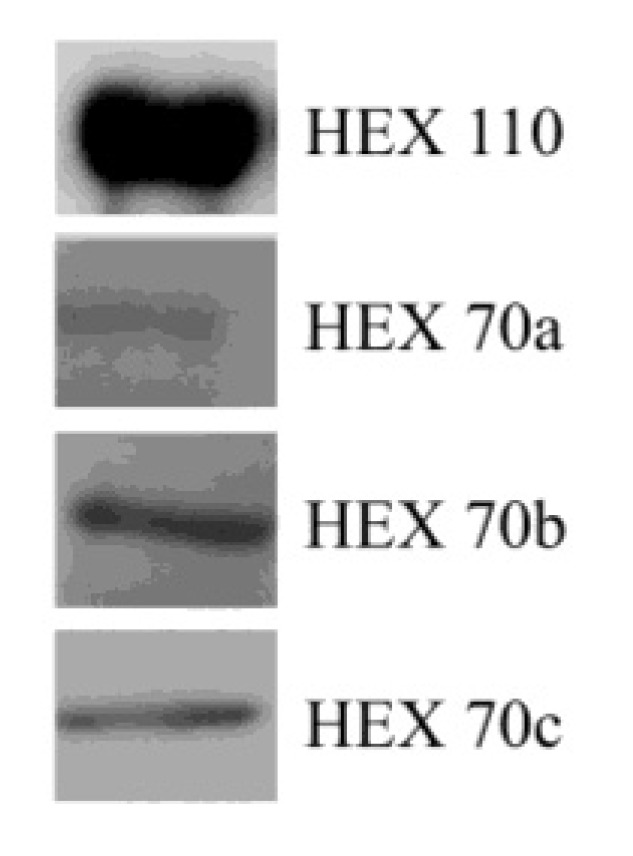
Hexamerins detected by western blot analysis in isolated fat body nuclei.

All hexamerins were detected in the nuclear fraction obtained from the fat body, as demonstrated by SDS-PAGE and western blot analysis using specific antibodies (Figure 2). 

### 3.3. Immunolocalization of Hexamerins in the Cytoplasm and Nucleus of Fat Body Cells

**Figure 3:**
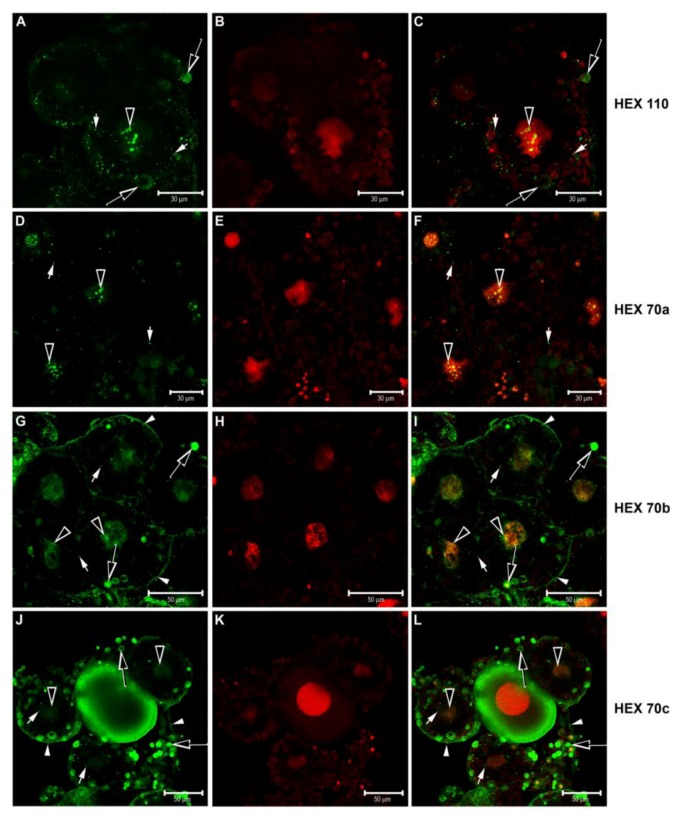
Confocal microscopy for detection of hexamerins in the trophocytes of pharate pupae (PP phase). Alexa Fluor 488-stained trophocytes: preparations with (**A**) anti-HEX 110, (**D**) anti-HEX 70a, (**G**) anti-HEX 70b and (**J**) anti-HEX 70c for detection of the respective hexamerins (green foci). (**B**, **E**, **H**, **K**) Propidium iodide-stained cell nuclei (red). At the right column, the merged images show: (**C**) HEX 110, (**F**) HEX 70a, (**I**) HEX 70b and (**L**) HEX 70c in the nuclei (yellow foci) and in the cytoplasm (green foci). Large hollowed arrowheads: nuclear foci of hexamerins. Small arrowheads: hexamerins in small granules near the cell surface. Large hollowed arrows: hexamerins in large cytoplasm granules. Small arrows: hexamerins in small cytoplasm granules. The cell in the center of Figures J, K and L is an oenocyte. The profusion of red inclusions in the trophocyte cytoplasm (**B**, **E**, **K**) are propidium iodide stained nucleic acids (DNA/ribosomal RNA), apparently derived from remnants of mitochondria and RER sequestered in autophagic vacuoles.

**Figure 4:**
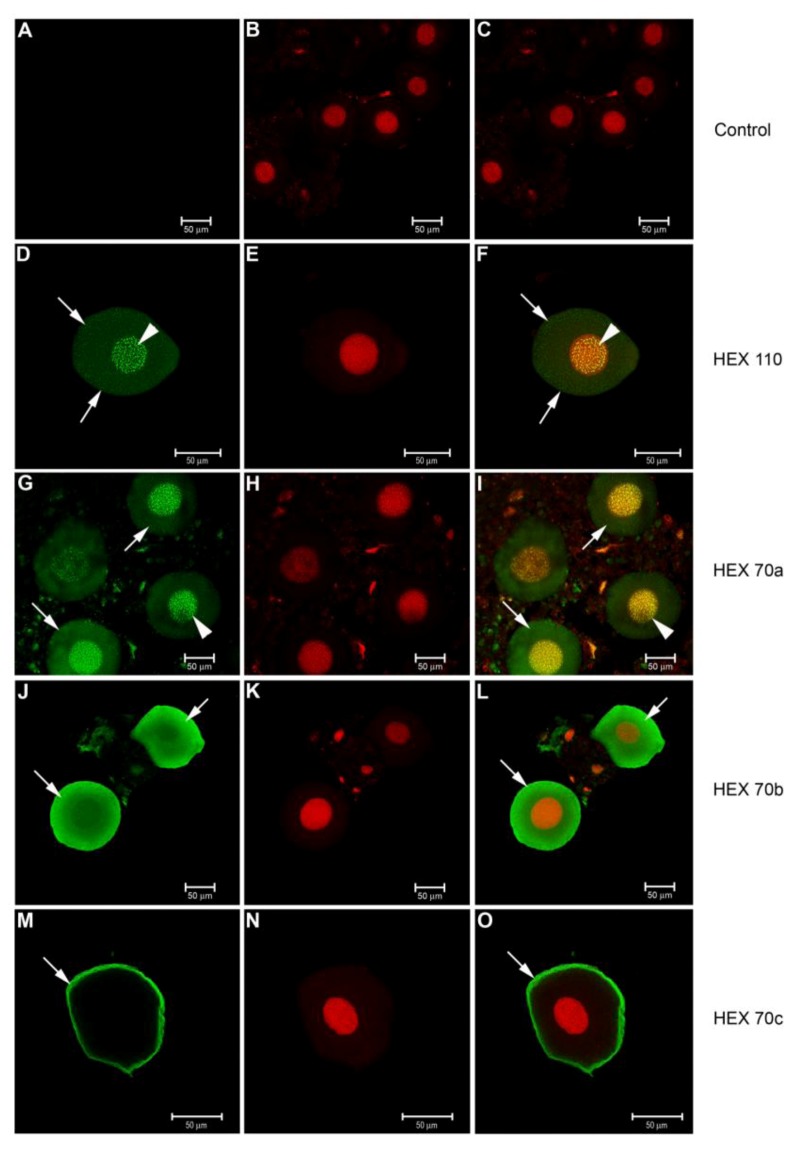
Confocal microscopy for detection of hexamerins in the fat body oenocytes of pharate pupae (PP phase). Alexa Fluor 488-stained enocytes: preparations (**A**) without the specific (primary) antibody (control) or with (**D**) anti-HEX 110, (**G**) anti-HEX 70a, (**J**) anti-HEX 70b or (**M**) anti-HEX 70c for detection of the respective hexamerins (green foci). (**B**, **E**, **H**, **K**, **N**) Propidium iodide-stained cell nuclei (red). At the right column, the merged images show: (**C**) the control without the antibody, (**F**) HEX 110 and (**I**) HEX 70a mainly in the nuclei (yellow foci) but also in the cytoplasm (green foci), (**L**) HEX 70b in the cytoplasm (green foci), (**O**) HEX 70c at the cytoplasmic periphery (green foci). Arrowheads: nuclear foci of hexamerins. Arrows: hexamerin foci in the cytoplasm.

We detected foci of all the honey bee hexamerins in the trophocytes (Figure 3) and oenocytes (Figure 4) in the fat body of pharate pupae (PP) by antibody staining and confocal microscopy. Preparations without the specific (primary) antibodies in Figure 4A–C are the “controls” of the immunolocalization procedures shown in Figure 3 and Figure 4. 

In the trophocytes, foci of HEX 110 (Figure 3A–C), HEX 70a (Figure 3D–F), HEX 70b (Figure 3G–I) and HEX 70c (Figure 3J–L) were evident in the nucleus and in the cytoplasm. The nuclear foci of HEX 110 (Figure 3A,C) and HEX 70a (Figure 3D,F) were more evident than the nuclear foci of HEX 70b (Figure 3G,I) and much more evident than the nuclear foci of HEX 70c (Figure 3J,L). The cytoplasmic foci of all four hexamerins were visualized as dense granules of different sizes or as large cytoplasmic inclusions, similar to the protein granules (heterophagic vacuoles) formed by fusing vesicles containing hemolymph proteins described by a previous study [12]. In the trophocytes shown in Figure 3G, we could see HEX 70b foci regularly localized at the cell surface. Hemolymph proteins have been described to concentrate at the cell surface before endocytosis occurs inside vesicles, which subsequently are separated from the cell membrane to form protein granules [12]. Interestingly, the trophocytes incubated with anti-HEX 70b (Figure 3G) and anti-HEX 70c (Figure 3J) show large granules of different densities near the cell surface, which suggests that they were derived from endocytosis of these hexamerins. 

A profusion of red inclusions were revealed in the cytoplasm of the trophocytes stained with propidium iodide (see Figure 3B,E,K and the respective merged images Figure 3C,F,L). Propidium iodide is a dye commonly used to visualize nuclear DNA, but it also binds to RNA. The red inclusions in the trophocyte cytoplasm apparently are autophagic vacuoles containing DNA and ribosomal RNA product remnants of mitochondria and RER recycling. At the time of protein granule formation in the pre-metamorphic fat body of *Calpodes ethlius*, cell organelles are isolated by membranes in autophagic vacuoles. RER-specific autophagic vacuoles may fuse with heterophagic vacuoles containing sequestered hemolymph proteins that form protein/RNA granules [12,35,36]. Similar to in *C. ethlius* [35], the red inclusions in the trophocyte cytoplasm (Figure 3B,E,H,K) are also characterized by the presence of an uncolored central region that matches the crystalline material in the center of the granules containing proteins and ribosomal RNA. 

Hexamerins were also localized in the oenocytes (Figure 4A–O). The oenocytes prepared without the specific (primary) antibodies in Figure 4A–C are the ‘controls’ of the immunolocalization procedure. The patterns of immunolocalization of HEX 110 (Figure 4D–F) and HEX 70a (Figure 4G–I) are similar, with evident foci of both proteins in the nuclei and smaller scattered foci in the cytoplasm. The other two hexamerins, HEX 70b (Figure 4J–L) and HEX 70c (Figure 4M–O), were localized exclusively in the oenocyte cytoplasm, and HEX 70c is limited to the peripheral cytoplasm.

### 3.4. Injection of Antibodies against Hexamerins in Pharate Adults: Effect on Survival and Timing to Adult Ecdysis

**Figure 5:**
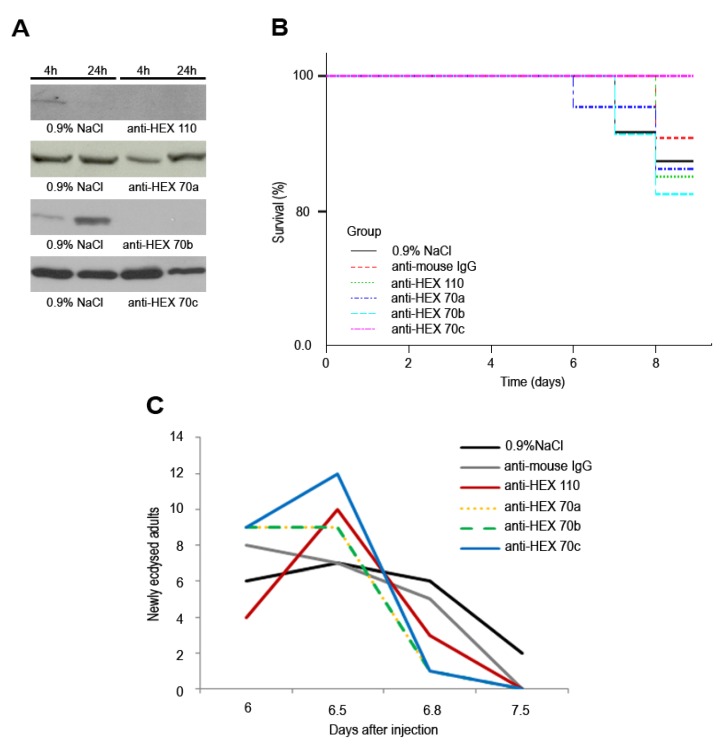
Effect of the injection of anti-hexamerins in pharate adults. (**A**) Western blots showing depletion of HEX 110, HEX 70a and HEX 70c in the hemolymph, and depletion of HEX 70b in the fat body of pharate adults, 4 h and 24 h after injection with 1 µg of the respective antibodies diluted in 0.9% NaCl. Control groups were injected with 0.9% NaCl or with 1 µg of mouse IgG in 0.9% NaCl. The western blot showing the effect of anti‑HEX 70a on HEX 70a depletion was previously shown (see Figure 4 in [30]). (**B**) All the bee groups (each containing 22 bees) showed similar survival rates, regardless of anti‑hexamerin injection (*p* = 0.524). (**C**) The injected and control groups significantly differed in the time elapsed to the adult ecdysis (*p* = 0.019). Data were analyzed using Kaplan-Meier survival analysis: Log-Rank followed by multiple comparison procedures (χ^2^ test) [37].

The injection of antibody against each hexamerin resulted in decreased levels of the corresponding protein in a proportion of pharate adults as shown by western blot analysis of the hemolymph or fat body. Noticeably, only a proportion of bees in each group, not exceeding 33%, effectively responded to the antibodies against hexamerins. Figure 5A shows representative samples taken from individual bees, which responded to the antibody by lowering the respective hexamerin level. This figure shows that 4 h after the injection of anti-HEX 110 or anti-HEX 70a, the levels of these hexamerins decreased in the hemolymph. However, normal levels of HEX 70a were recovered 24 h after the injection. A decrease in the hemolymph levels of HEX 70c was observed 24 h after the injection (and maintained up to 129 h, data not shown). Because the level of HEX 70b is normally very low in the hemolymph at the time of the antibody injection (Pw phase, see Figure 1), and thus impairs the analysis of the effect of anti-HEX 70b, we used the fat body (where the HEX 70b level is still high, see Figure 1) in the western blot analysis shown in Figure 5A. This figure illustrates that the injection of the antibody caused HEX 70b depletion in the fat body 4 h after the injection. This was confirmed at 24 h (Figure 5A) and at 129 h (data not shown). This last time point precedes the pharate adult phase (Pbm phase), characterized by lowering levels of some of the hexamerins in the hemolymph (see Figure 1).

The injections did not significantly affect survival (*p* = 0.524) up to adult ecdysis (Figure 5B), but the bees injected with the antibodies against hexamerins underwent adult ecdysis significantly earlier than the controls injected with 0.9% NaCl or mouse IgG (*p* = 0.019) (Figure 5C).

## 4. Discussion and Conclusions

During the metamorphic molt in holometabolous insects, the fat body changes its typical larval architecture, made of layers or clumps of cells, to dissociated individual cells that lose their adhesion to each other [38]. The fat body cells are mostly free in the hemolymph of the honey bee at the pharate pupae stage [39,40]. At the following pupal and early pharate adult stages, there is a switch from synthesis and secretion of hexamerins to their sequestration. Studies establishing the correlation between the depletion of hexamerins from the hemolymph and the appearance of granules in the trophocyte cytoplasm predicted that the sequestered hexamerins are stored in these granules for further utilization [12,13]. This dynamic swap was apparent when we compared the levels of HEX 70b in the hemolymph and fat body of metamorphosing honey bees. There was a progressive decrease of HEX 70b in the hemolymph of the pharate pupa (PP phase), pupa (Pw phase) and early pharate adult (Pb phase) as it increased in the fat body of these same bees (see Figure 1). Note that the dynamic swap was investigated in the same bee, and not in pooled bees, to prevent potential interferences provoked by temporal differences within the same developmental phase. HEX 70c also showed a similar dynamic, although not as distinctly. The HEX 110 behavior was somewhat different because we could not see a clear increase of this hexamerin in the fat body. Like the majority of the hexamerins, HEX 70b, HEX 70c and HEX 110 are essentially ‘metamorphosis proteins’, and as such, their abundance lowers to basal levels at adult ecdysis. However, as reported previously [25,28], HEX 70a persists even in adults (see Figure 1). Based on the contrasting expression profiles of the fat body and hemolymph, HEX 70b may be considered as the most stereotypical hexamerin in the honey bee.

Hexamerin foci were detected in small and large spheroid inclusions, similar to protein granules of different sizes, in the cytoplasm of the trophocytes from pharate pupae. The appearance of protein granules in the fat body of *A. mellifera* shortly before pupation was reported decades ago by Bishop [41,42]. Using schematic illustrations, Snodgrass [39] referred to the localization of these granules in the periphery of the trophocyte cytoplasm in the honey bee pharate pupae and also commented that the number of these protein granules continues to increase during the early part of the pupal stage, and then disappears near the time of adult ecdysis. Poiani and Cruz-Landim [43] also found large amounts of protein granules in the trophocytes from pharate pupae. The spheroid inclusions, or granules, stained with anti-HEX 70c and with anti-HEX 70b in the trophocytes were mainly localized at the cytoplasmic periphery (see Figure 3G,I,J,L), similar to the protein granules described by Snodgrass [39]. Apparently, the observed granules are involved in the storage and recycling of HEX 70b and HEX 70c. Granules stained with anti-HEX 110 and anti-HEX 70a were less abundant in the trophocytes, but this may be due to fat body regionalization. In *D. melanogaster*, the rate of protein granule formation differs among regions of the fat body [44]. Because we used dissociated fat body from pharate pupae whole body, the trophocytes shown in Figure 3 may come from different regions. Small protein granules are initially formed in the trophocytes and grow by fusing with other granules [12]. It is then possible that the fusion of protein granules is just starting in the trophocytes shown in Figure 3A (stained with anti-HEX 110) and 3D (stained with anti-HEX 70a).

The presence of hexamerins in the cytoplasm of the trophocytes was expected, but we also found small foci of hexamerins in the oenocyte cytoplasm. Oenocytes differ from trophocytes in embryological origin, morphology, and in biochemical and physiological functions; they are required for lipid processing, larval growth, the production of pheromone and developmental signaling [38,45]. Because storage proteins were up to now described in the trophocytes, which are the cells that accumulate proteins in granules and cell inclusions, it was surprising to find hexamerins in the oenocytes. Even more remarkable was to detect the four hexamerins in the nuclei of the trophocytes and HEX 110 and HEX 70a in the nuclei of the oenocytes.

The immunolocalization of the hexamerins in the fat body cell nuclei confirmed the western blot results (Figure 2), which revealed the presence of the four hexamerins in fat body nuclear-enriched fractions. 

Previously, our laboratory showed that HEX 70a is localized in the nuclei of ovarian and testis cells, which implied a novel role for this hexamerin in the gonads. The nuclear colocalization of HEX 70a with the cell cycle S-phase marker EdU further indicated that HEX 70a may play a role in DNA replication for cell proliferation or polyploidization [30]. This hypothesis is supported by previous experiments demonstrating that a fat body hexamerin (a 77 kDa arylphorin subunit) in lepidopterans was efficient in stimulating the *in vitro* proliferation of larval midgut stem cells. Furthermore, experiments using BrdU labeling confirmed that arylphorin induces DNA synthesis and has mitogenic-stimulating activity *in vivo* [46,47,48,49,50,51].

However, in contrast to the honey bee ovarian cystocytes, which are mitotically active as shown by BrdU (5-bromo-29deoxy-uridine) labeling [52] and also by EdU labeling [30], the trophocytes in the pharate pupae fat body are not engaged in cell division, but rather in cell dissociation for metamorphosis. Therefore, the function of hexamerins in the fat body nucleus of pharate pupae is certainly not related to cell proliferation. Interestingly, a storage protein (SP2) from the hemolymph of *Bombyx mori*, which shows 25%–30% similarity with the honey bee hexamerins, suppressed nuclear fragmentation and apoptotic body formation in HeLa cell cultures [53]. This intriguing result may indicate that at the pharate pupae stage, the hexamerins protect the fat body cells from cell death during metamorphosis. 

This dual function, regulation of cell proliferation and protection against apoptosis, has been demonstrated for a protein kinase, CK2, which has long been linked to cell proliferation, but which also acts as an anti-apoptotic protein in cancer cells [54]. CK2 inactivates proteins involved in promoting apoptosis, and has been associated to cell survival through the regulation of the function of proteins with roles in transcription, cell signaling, cell-cycle control and DNA repair [55,56]. In the same way it was inferred for hexamerins, CK2 translocates from the cell cytoplasm to the nucleus. Putative substrates for CK2 in the nucleus may include growth factors and non-histone proteins, including transcript factors; chromatin as well as the nuclear matrix should be the preferential targets for CK2 association in the nucleus [54]. 

Our previous data [30] strongly suggested a function of hexamerins in gonadal cell proliferation. The current data allow us to suggest a complementary role of nuclear hexamerins in protecting fat body cells from cell death during the metamorphic transition. It is noteworthy that few trophocytes in our histological preparations were labeled with anti-HEX/Alexa fluor, being the enocytes labeled more frequently. Labeled cells may be those that were preserved from apoptosis. 

Supporting our data indicating the presence of hexamerins in cell nucleus, Begna *et al.* [57] also detected HEX 110 in the nuclear proteome of worker and queen honey bee larvae in the fourth and fifth instars by using nuclear protein enrichment, two-dimensional electrophoresis and mass spectrometry.

Here, we have also shown that the injection of each anti-hexamerin antibody at the working concentration of 1 µg in the hemocoele of early pharate adults may cause partial or total depletion of hexamerins. The injections of anti-hexamerins did not result in significant mortality in comparison to the control groups. However, the timing of pharate adult development and the ecdysis to the adult stage were significantly accelerated. By cross-reacting with hexamerins, and hence inactivating them, the injected antibodies may have disturbed the utilization of these proteins for pharate adult development. Interestingly, a precocious ecdysis was observed independently of the type of the injected anti-hexamerin antibody. In spite of the differences in the effectiveness of the knockdowns, resulting in total or partial depletion of the hexamerins, or even depletion followed by recovery to normal levels, all the anti-hexamerin-treated bees ecdysed earlier than the controls. Though consistent, this result is difficult to explain, and the complexity increases even more when it is considered that in the hemolymph, the subunits may combine to form a heteromeric hexamerin. If so, by cross-reacting with the specific subunit in the heteromer, the antibody also indirectly precipitates non-specific subunits. This would tentatively explain the similar effect of injection of each specific antibody on ecdysis timing. In this context, further studies on the interaction of the subunits and structural organization of hexamerins are relevant.

While pupae and pharate adults do not feed, their development is supported by the endogenous stocks of nutrients accumulated during the larval stage. Hexamerins are the main storage proteins in the larval hemolymph and the main source of amino acids for pupal and pharate-adult development. Functioning as nutritional sensors, the natural depletion of hexamerins may play a critical role in signalizing the onset of adult ecdysis. It is possible that the decrease in soluble hexamerins in antibody-treated pharate adults induced a precocious adult ecdysis in response to the lack (or decrease) in hexamerin-derived amino acids. 

It is known that metamorphosis in insects is initiated by an as yet largely unknown size-sensing mechanism [58], where the nutritional status of the larvae has an important role in controlling its final size and the onset of the metamorphic molt. Entry into the metamorphic molt depends on a size threshold, or critical weight, at which endocrine events lead to cessation of feeding and onset of metamorphosis [59,60]. We hypothesize that a nutrient-sensing signal also functions in pharate adults, thus controlling developmental timing to adult ecdysis, which may be disturbed by the depletion of storage proteins. This hypothesis requires further investigation.

Taken together, our results enhance the understanding of the functions of hexamerins. Clearly, the combined use of molecular biology, genetic and biochemical tools are needed to specify the nuclear function of hexamerins in the fat body during insect development. 
